# Is plant-based protein supplementation as effective as whey protein? A systematic review and meta-analysis in untrained adults undergoing resistance training

**DOI:** 10.1080/15502783.2026.2752317

**Published:** 2026-10-29

**Authors:** Irineu Otavio Marchiori Callegari, Giovana Molina Silva, Alexandre Gabarra Oliveira

**Affiliations:** a Department of Physical Education, Institute of Biosciences, São Paulo State University (UNESP), Rio Claro, Brazil

**Keywords:** Protein synthesis, body composition, muscle hypertrophy, nutrient supplementation, protein intake

## Abstract

**Background:**

Dietary protein supplements are commonly consumed to support increases in lean body mass among healthy adults engaged in resistance training (RT). Although whey protein has been extensively investigated, plant-based protein supplements have emerged as a viable alternative for individuals who avoid animal-derived products.

**Methods:**

The present study aims to evaluate, through a systematic review and meta-analysis, the effects of plant-based versus whey protein supplementation on body composition in untrained adults (18–59 years) undergoing RT, using Dual-Energy X-Ray Absorptiometry (DEXA). The systematic review was conducted according to the Preferred Reporting Items for Systematic Reviews and Meta-Analyses (PRISMA) 2020 checklist and was registered in PROSPERO (CRD420251145410). Searches were conducted in PubMed, Web of Science, and ScienceDirect, identifying 5 studies that met the inclusion criteria for qualitative synthesis and meta-analysis.

**Results:**

When combined with RT, both plant-based and whey protein supplementation were associated with improvements in lean body mass (LBM) and body fat mass (BFM), with no significant between-group differences observed.

**Conclusions:**

Thus, soy- and pea-based protein supplements may support favorable body composition changes in adults initiating RT and could be considered practical nutritional options for individuals seeking alternatives to whey protein. Regarding chia-based protein, the available evidence is currently limited to a single study that did not demonstrate significant changes in lean body mass; therefore, firm conclusions regarding its efficacy cannot be drawn at this time.

## Key points

- This systematic review and meta-analysis suggest that both plant-based and whey protein supplementation may support improvements in LBM and BFM in untrained adults undergoing resistance training, with no statistically significant between-group differences observed.
- When matched for total protein intake and training variables, the source of protein – plant or animal – did not result in statistically significant differences in hypertrophic or body composition outcomes, though the limited number of studies and wide confidence intervals preclude definitive conclusions.
- These results highlight the potential of plant-derived proteins as a sustainable nutritional alternative to whey protein, which may support favorable body composition adaptations under controlled conditions.
- The present findings suggest that the traditional assumption of whey protein’s superiority may warrant re-examination; however, the current evidence is insufficient to establish physiological equivalence, and further adequately powered trials are needed before definitive claims can be made.
- When resistance training protocols and total protein intake are rigorously controlled, plant-based proteins did not show a statistically significant difference from whey protein in the present analysis; however, this finding should not be interpreted as evidence of physiological equivalence, and the limited number of available studies and wide confidence intervals prevent firm conclusions at this time.

## 1. Introduction

An increase in lean body mass and muscular strength is a highly desired outcome among individuals engaged in resistance training (RT). However, when daily protein intake targets are not achieved through diet alone, the potential adaptations to RT may be attenuated. Current protein recommendations suggest an intake of 0.8 g/kg of body weight for healthy young adults, increasing to about 1.2 g/kg of body weight in older adults, and 1.6–1.8 g/kg in athletes [1]. In this regard, Morton and colleagues conducted a systematic review demonstrating that these protein intake levels are optimal for promoting gains in muscle mass under RT conditions [2]. Consequently, there has been increasing interest in high-protein supplementation not only among athletes but also within the general population, due to its potential to enhance body composition and contribute to the prevention of sarcopenia [3–5].

In this context, dietary protein sources of both animal origin (e.g. whey, casein, beef) and plant origin (e.g. soy, pea, or rice) are widely available and have been extensively investigated. Most of these protein sources have been shown to effectively stimulate post-exercise myofibrillar protein synthesis rates [6]. However, whey protein supplementation (WPS) is particularly popular due to its high digestibility in most adults and its capacity to rapidly deliver amino acids to skeletal muscles, thereby facilitating the fulfillment of the body’s protein requirements. WPS is often regarded as the most effective supplement for stimulating protein synthesis, primarily attributed to its high leucine content, an amino acid known to activate the mechanistic target of rapamycin complex 1 (mTORC1) to a greater extent than casein, for example [1].

However, recent evidence has demonstrated that well-formulated plant-based protein supplement (PPS) blends, (for example, combinations of pea, rice, and canola) can also stimulate muscle protein synthesis to levels comparable to whey protein when consumed in adequate doses (≥30 g with ~2.5 g of leucine) [7]. This positions plant-based proteins as a viable nutritional alternative to animal-based proteins—a strategy increasingly adopted in sports nutrition practice [8] and supported by a growing body of evidence suggesting comparable benefits for lean mass accretion and body fat maintenance when total protein intake and training variables are adequately controlled [9,10]. Additional motivations for the use of plant-based protein sources include reduced environmental impact, lower demand for livestock production, and decreased prevalence of food intolerances, particularly those related to lactose and casein [11]. Nevertheless, more long-term, high-quality clinical trials in vegetarian and vegan populations are still required to establish definitive recommendations for the use of plant-based protein in sports nutrition.

In addition to muscle hypertrophy, characterized by an increase in muscle mass and strength, individuals engaging in RT also seek improvements in overall body composition, particularly through the reduction or maintenance of body fat mass (BFM) and increase of lean body mass (LBM), which is often used interchangeably with muscle mass [12]. RT consists of exercises employing external loads that generate mechanical tension in skeletal muscle, which, when leading to hypertrophy, may result in increased body weight not necessarily accompanied by higher body fat percentage, thus contributing positively to body composition [13].

The nuances described above make the comparison of protein sources and their impact on RT adaptations a highly debated topic, prompting ongoing scientific discussion. Accordingly, the objective of this systematic review and meta-analysis was to evaluate the effects of PPS compared with WPS on the body composition of healthy adults with no prior experience in resistance exercise. We hypothesized that, although WPS has stronger scientific support, PPS may produce comparable effects, potentially promoting muscle hypertrophy and reductions in body fat percentage to a similar extent in adults undergoing RT.

## 2. Methods

This study was conducted in accordance with the Preferred Reporting Items for Systematic Reviews and Meta-Analyses (PRISMA) 2020 guidelines [14] and was prospectively registered in PROSPERO (Center for Reviews and Dissemination, University of York; http://www.crd.york.ac.uk/PROSPERO) under the registration number CRD420251145410.

### 2.1. Search strategy

Searches were conducted in PubMed (*n* = 43 records), Web of Science (*n* = 8 records), and ScienceDirect (*n* = 19 records), totaling 70 records (Figure 1), using the following keywords strategy:

**Figure 1.**
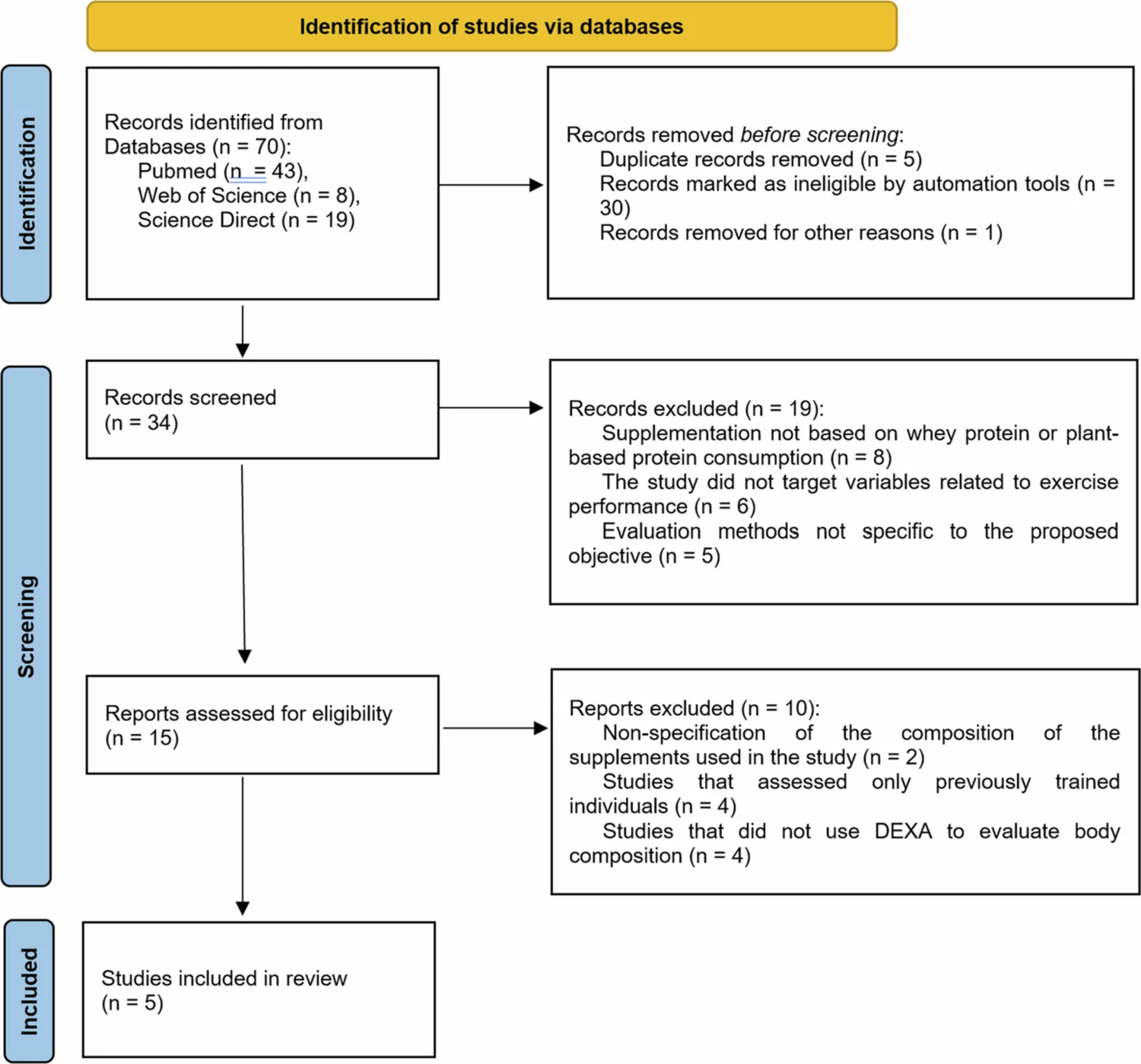
Preferred Reporting Items for Systematic Reviews and Meta-Analyses (PRISMA) flow diagram for identification of studies via databases.

(1) (muscle mass[Title/Abstract] OR hypertrophy[Title/Abstract] OR muscle enlargement[Title/Abstract] OR skeletal muscle enlargement[Title/Abstract] OR muscle gain[Title/Abstract]) AND (whey protein[Title/Abstract] OR isolate Whey protein[Title/Abstract] OR concentrated Whey protein[Title/Abstract] OR hydrolyzed whey protein[Title/Abstract]).
(2) (vegan protein[Title/Abstract] OR vegetarian protein[Title/Abstract] OR plant based protein[Title/Abstract] OR plant protein[Title/Abstract] OR rice protein[Title/Abstract] OR soy protein[Title/Abstract] OR pea protein[Title/Abstract]) AND (muscle mass[Title/Abstract] OR hypertrophy[Title/Abstract] OR muscle enlargement[Title/Abstract] OR skeletal muscle enlargement[Title/Abstract] OR muscle gain[Title/Abstract]).

Randomized controlled trials published in English within the last 20 years (February 2005 to February 2025) were considered.

The search was conducted independently in each database, with the following dates and filters applied: PubMed (searched April 2025; filters: Randomized Controlled Trial, Humans); Web of Science (searched July 2025; no filters applied); and ScienceDirect (searched July 2025; filters: publication years 2005–2025, article type restricted to research articles).

Due to clinical and methodological variability, this meta-analysis included only studies that assessed body composition using Dual-Energy X-Ray Absorptiometry (DEXA) in adults (18 to 59 years old) who were untrained in resistance exercise. This restriction was intentionally adopted to minimize methodological heterogeneity and improve internal validity, since alternative body composition methods (e.g. bioelectrical impedance analysis, skinfold thickness, or anthropometric equations) differ substantially from DEXA in precision and sensitivity for detecting small changes in lean and fat mass. The PRISMA 2020 flow diagram for new systematic reviews, including database and register searches [14], is presented in Figure 1:

### 2.2. Inclusion criteria

To be included in the review, studies were required to meet the following criteria, based on the PICOS framework (Population, Intervention, Comparison, Outcomes, Study design):

P—participants: healthy men and women aged 18–59 years;

I—intervention: consumption of high-protein dietary supplements based on plant proteins (e.g. soy, wheat, pea, rice);

C—comparison: group receiving high-protein dietary supplements based on whey protein;

O—outcomes: studies assessing lean and/or muscle mass and body fat separately;

S—study design: randomized clinical trials lasting at least six weeks.

The studies included in this meta-analysis also met the following additional criteria: (a) randomized controlled trials; (b) intervention groups receiving whey protein in isolated, concentrated, or hydrolyzed forms, rather than multicomponent supplements or protein-rich foods such as milk; (c) participants aged 18-59 years; (d) intervention periods lasting at least six weeks or more in RT; (e) inclusion of a whey protein supplementation arm as the active comparator to the plant-based protein group, with a subset of studies additionally including a placebo or non-supplemented control arm not used in the present PPS versus WPS comparison; (f) body composition assessed using DEXA, including lean body mass and fat mass; (g) availability of total caloric intake data before and after the intervention; (h) means/medians and standard deviations (SD), standard error (SE) or interval values reported for outcomes of interest; and (i) participants without a recent history of high-protein supplement intake. When multiple time points were reported, the means difference (MD) and SD from the most recent measurement were used to avoid data duplication.

Studies that did not meet all predefined inclusion criteria were excluded from this review, as illustrated in Figure 1.

### 2.3. Data extraction and quality control

Two authors (IC and GM) independently screened the titles and abstracts of all citations identified during the systematic search. After initial screening, extracted data were compared between the two researchers to ensure completeness and reliability. All selected studies were then independently evaluated against the inclusion criteria. In cases of uncertainty or disagreement regarding inclusion or exclusion, the final decision was made by a third author (AO).

During screening and subsequent review, the following data were extracted from each included study: first author, publication year, participant characteristics, intervention and control types, study duration, blinding type, sample size per group, supplement type and dosage, structure and design of the resistance training program, and exercise selection. Mean difference (MD) and standard deviation (SD) or interval values for body composition indicators were also extracted.

Two authors (IC and AO) assessed the methodological quality of each included study and evaluated the risk of bias using the Cochrane Risk of Bias 2 tool (RoB2, 2019) [15]. The assessment considered five domains: (1) bias arising from the randomization process; (2) bias due to deviations from intended interventions; (3) bias due to missing outcome data; (4) bias in measurement of the outcome; and (5) bias in selection of the reported result. Each domain was judged as low risk of bias, some concerns, or high risk of bias according to the RoB2 criteria. Disagreements between reviewers were resolved through discussion and, when necessary, consultation with a third author. The risk-of-bias assessments were summarized and graphically presented using the robvis package in R software.

### 2.4. Statistical analysis

A meta-analysis was conducted when at least three studies compared the effects (pre- and post-intervention data) of PPS versus WPS for a given endpoint. The pooled mean difference (MD) between interventions (post- minus pre-intervention data) and corresponding 95% confidence intervals (CI) were calculated using a random-effects model with restricted maximum likelihood (REML) as the heterogeneity variance estimator. Continuous outcomes were pooled using the metacont function of the R meta package based on the reported group means and standard deviations, without requiring imputation of the change-score standard deviation from an assumed pre–post correlation. Publication bias was not formally evaluated using funnel-plot-based tests (e.g. Begg’s or Egger’s tests), as these methods have limited statistical power when fewer than ten studies are included per outcome and may produce misleading results. Heterogeneity among studies was assessed using the Q statistic and I^2^ index, with I^2^ values of 25%, 50%, and 75% interpreted as low, moderate, and high heterogeneity, respectively. All statistical analyses were performed using the *meta* package of R software, version 4.5.1 (2025-06-13 ucrt) [16].

## 3. Results

### 3.1. Study selection

A total of 70 records were identified across the three databases explored (PubMed *n* = 43, Web of Science *n* = 8, ScienceDirect *n* = 19). Following removal of duplicates and records excluded by automation tools or for other reasons prior to screening (*n* = 36), 34 records were screened by title and abstract, of which 19 were excluded for not meeting the PICOS inclusion criteria of this meta-analysis. In addition, 10 more studies were excluded due to methodological ineligibility, including lack of specification regarding supplement composition, studies involving previously trained individuals, or those that did not use DEXA to assess body composition, as shown in Figure 1, which illustrates the final selection and exclusion process. Ultimately, 5 studies met all predefined criteria for inclusion in the meta-analysis [17–21]. Table 1 provides a detailed description of all studies included in the analysis.

**Table 1.** Description of studies included in the meta-analysis.

	Study (year)	Experimental design	Subjects	Age	Supplement type	Dose used	RT intervention	RT protocol	Reported outcomes	Main conclusion
**1**	**Zbinden-Foncea et al. [17]**	Randomized, controlled, single-blinded study	80 young men, healthy, untrained	19-25 years	Chia Protein vs. Whey Protein Concentrate	50 g chia flour containing 20 g protein	3x/week, 8 weeks	Whole-body resistance training	Strength tests (lower- and upper-limb one repetition maximum (1-RM) tests) and body composition analyses (DEXA)	Neither chia flour nor WPS elicited an enhanced effect on LBM and strength consuming a habitual high protein mixed diet (>1.2 g/kg/day)
						30 g whey protein concentrate containing 23 g protein				
**2**	**Mobley et al. [18]**	Double-blinded and placebo-controlled	75 young men, healthy, untrained	19-23 years	Whey protein concentrate vs. Whey protein hydrolysate vs. Soy protein concentrate	Formulated to provide ~3 g of leucine, per serving	3 × /week, 12 weeks	Free-weighted resistance training	Skeletal muscle and subcutaneous fat biopsies. Total body skeletal muscle mass determined by DEXA. Maximal force production capacity was assessed using an isometric mid-thigh pull test, body strength was assessed using a 3-RM squat and bench press.	Protein supplementation does not provide added benefit in increasing skeletal muscle mass or strength. WPS increases skeletal muscle satellite cell number.
**3**	**Volek et al. [19]**	Randomized double-blind, parallel clinical trial	72 non-resistance-trained adults (men and women)	18-35 years	Soy Protein isolate vs. Whey Protein Concentrate	The daily protein supplement (∼22 g.day−1) increased protein intake to ∼1.4g .kg−1	9 months	whole-body periodized resistance training	Body composition was assessed using DEXA. Performance testing consisted of 1-RM squat and bench press. Blood samples were obtained for plasma analysis.	WPS was more effective than soy protein treatment conditions in promoting gains in LBM.
**4**	**Singh et al. [20]**	Randomized triple-blind, comparator-controlled, parallel clinical trial	84 sedentary adults (men and women)	30-59 years	Pea Protein vs. Whey protein isolate	20g pea protein vanilla and 22.5g pea protein chocolate. Whey protein vanilla contained 20g and 21.1g chocolate	30min/session, 6x/week. 12 weeks (84 days)	Resistance training, alternating days between upper and lower limbs	The knees and elbow flexors and extensors movements using an isometric dynamometer. The handgrip strength using a hand dynamometer. Treadmill submaximal walking test. Waist circumference and body composition using DEXA	Pea Protein supplementation resulted in improvements in strength and LBM comparable to WPS when combined with a resistance training
**5**	**Lynch et al. [21]**	A prospective, two-group parallel-arm, randomized, double-blind study	61 untrained young adults (men and women)	18-35 years	Whey Protein Isolate vs. Soy Protein Isolate	19 grams of whey protein isolate or 26 grams of soy protein isolate, both containing 2 g (grams) of leucine	3 × /week, 12 weeks	whole-body periodized resistance training	1-RM weight lifted was calculated for the bench press, leg press, and knee extensions. Muscle thickness of the dominant leg was assessed using ultrasound. Body composition was assessed using DEXA. Muscle strength and torque peak was determined for the leg extensors and flexors on an isokinetic dynamometer.	Increases in LBM and strength in untrained participants are comparable when strength training and supplementing with soy or whey matched for leucine

DEXA = Dual-Energy X-Ray Absorptiometry; LBM = lean body mass; BFM = body fat mass; RT = resistance training; WPS = whey protein supplementation; PPS = plant protein supplementation; RM = repetition maximal test.

### 3.2. Study population, supplementation, and training protocols

Data from 372 participants aged 18-59 years were included in the evaluation of WPS versus PPS. Two studies recruited only male participants [17,18], while the remaining three studies recruited both men and women [19–21].

All included studies featured experimental groups receiving WPS and PPS, with whey protein supplementation serving as the active comparator to plant-based protein supplementation in all studies; only one study [18] additionally included a placebo-controlled arm, which was not used in the present PPS versus WPS comparison. Among PPS interventions, two studies used isolated soy protein [19,21], one used soy protein concentrate [18], one used pea protein [20] and one used chia-based protein [17]. Regarding WPS formulations, two studies employed whey protein isolate [20,21], while the remaining three used whey protein concentrate for the calculation of the reported outcomes [17–19]. It was not possible to select studies with identical supplementation dosages across trials, as shown in Table 1.

The resistance training protocols were carefully reviewed in this study and considered a key inclusion criterion. All included studies implemented RT protocols using external loads, including Mobley et al. [18], Lynch et al. [21], Volek et al. [19], Zbinden-Foncea et al. [17], and Singh et al. [20]. Three studies adopted whole-body training sessions [17,19,21], whereas one alternated upper- and lower-body sessions [20]. Strength assessment was incorporated to appropriately adjust training loads throughout the interventions. Singh et al. [20] evaluated isometric strength using a dynamometer, Lynch et al. [21] used 1-RM testing and isokinetic dynamometry, while the remaining studies used one-repetition maximum (1-RM) testing [17–19].

### 3.3. Risk of bias assessment

As shown in Table 2, the overall methodological quality of the included studies was considered moderate to high. Most studies presented low risk of bias for randomization, intervention deviations, and outcome assessment due to the use of randomized and blinded designs combined with objective body composition measurements (DEXA). However, some studies raised concerns regarding allocation concealment, missing outcome data, and selective reporting, resulting in an overall judgment of ‘some concerns’ according to the RoB2 tool.

**Table 2.** Risk of bias assessment of the included randomized controlled trials using the Cochrane Risk of Bias 2 (RoB 2) tool.

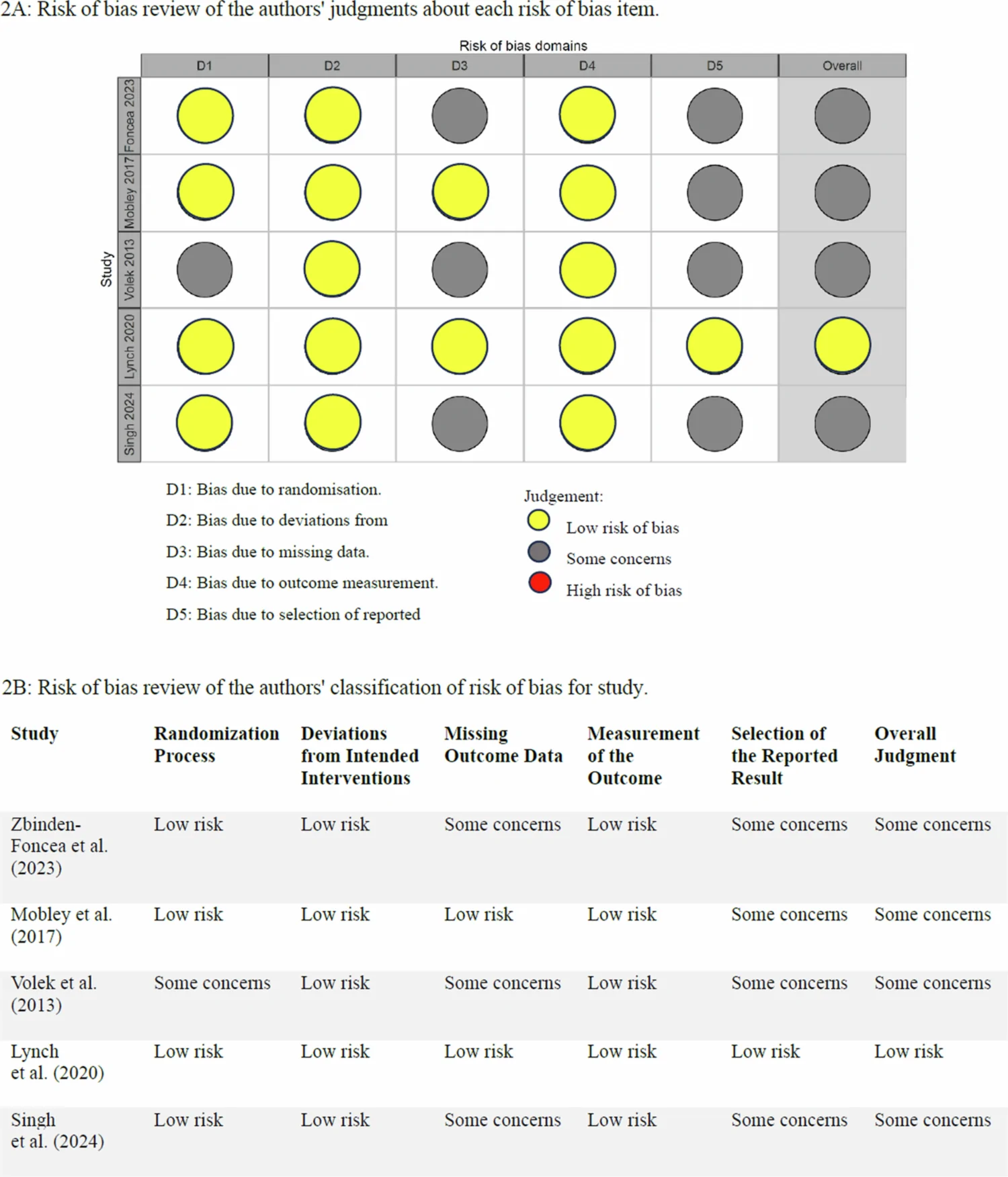

### 3.4. Body composition

Overall, no significant differences were observed in LBM changes between WPS and PPS groups (Figure 2), as all confidence intervals of mean differences included zero. Heterogeneity across studies was also low (I^2^ = 0%, τ^2^ = 0, *p* = 0.8819). The weights assigned to each study were derived from the inverse of the total variance (within-study variance plus the between-study variance, τ^2^, under the random-effects model), reflecting each study’s precision and its relative contribution to the pooled effect estimate.

**Figure 2.**
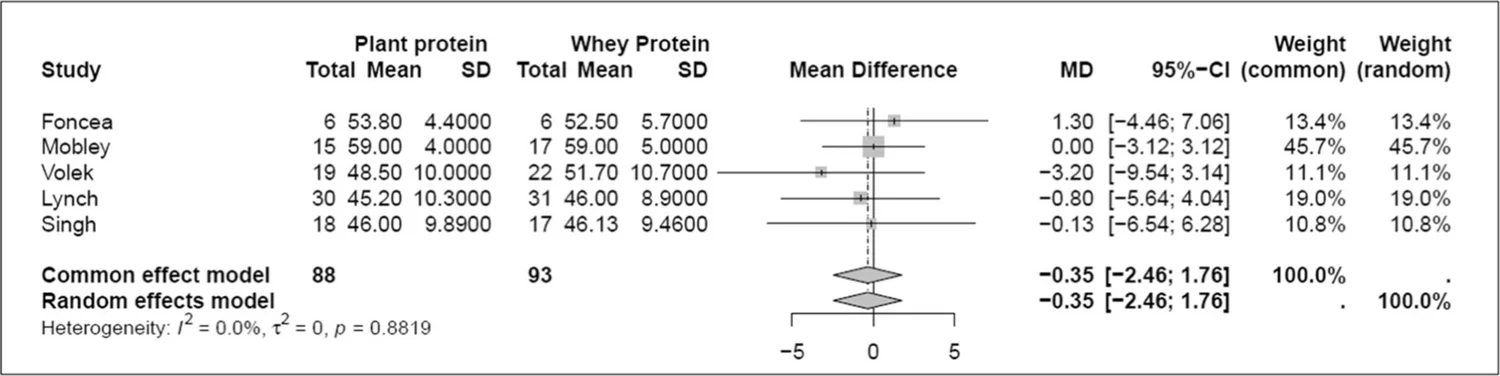
Forest plot showing the effect of protein source supplementation (plant protein vs. whey protein) combined with RT, on change (MD) in LBM. The τ^2^ value represents the estimated heterogeneity between studies. LBM = lean body mass; RT = resistance training; MD =  mean difference; SD = standard deviation; CI = confidence interval.

Mobley et al. [18] reported a mean difference near zero (MD = 0.00; 95% CI: −3.12 to 3.12), with the confidence interval including zero and suggesting no statistically significant effect. Zbinden-Foncea et al. [17] demonstrated a positive trend (MD ≈ 1.30; 95% CI: −4.46 to 7.06), though its wide confidence interval also encompassed zero, indicating inconclusive evidence. Volek et al. [19] showed the largest negative mean difference (MD ≈ −3.20; 95% CI: −9.54 to 3.14), with a wide confidence interval also crossing zero. Finally, Singh et al. [20] (MD ≈ −0.13; 95% CI: −6.54 to 6.28) and Lynch et al. [21] (MD ≈ −0.80; 95% CI: −5.64 to 4.04), as well as the pooled estimate across studies (MD = −0.35; 95% CI: −2.46 to 1.76), presented negative or near-zero mean differences with confidence intervals crossing zero, reinforcing the absence of consistent effect on LBM.

Regarding BFM, the meta-analysis revealed no significant heterogeneity among the four included studies (I^2^ = 0%, τ^2^ = 0), indicating consistent effects across studies (Figure 3). Zbinden-Foncea et al. [17] reported a non-significant mean difference in BFM between PPS and WPS (MD ≈ −2.90; 95% CI: −7.81 to 2.01). Similarly, the remaining three studies presented confidence intervals encompassing zero (Mobley et al. [18]: 95% CI −2.47 to 4.47; Lynch et al. [21]: 95% CI −3.93 to 3.13; Volek et al. [19]: 95% CI −5.84 to 8.04), confirming the absence of a statistically significant effect on BFM. In summary, PPS and WPS produced comparable effects on BFM, with no evidence supporting the superiority of either intervention over the other.

**Figure 3.**
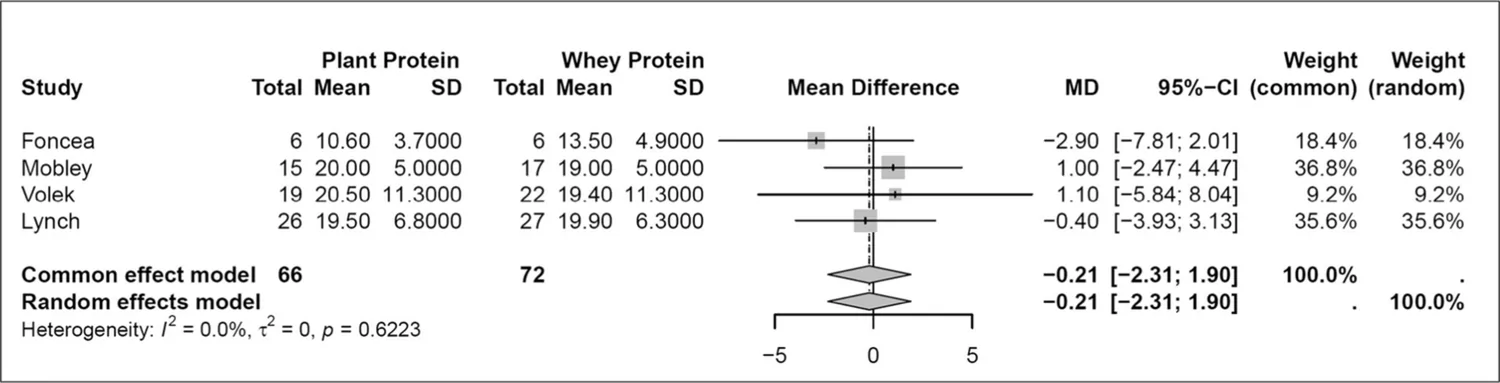
Forest plot showing the effect of protein source supplementation (plant protein vs. whey protein) combined with RT, on change (MD) in BFM. The τ^2^ value represents the estimated heterogeneity between studies. BFM = body fat mass; RT = resistance training; MD =  mean difference; SD = standard deviation; CI = confidence interval.

## 4. Discussion

The objective of this systematic review and meta-analysis was to compare the effects of WPS *versus* PPS on body composition in untrained adults. Although high-protein supplements are widely used, the current study specifically focused on outcomes related to improvement in body composition. In this context, cultural, ethical, and environmental trends have increasingly encouraged the reduction of animal-derived product consumption and the adoption of plant-based alternatives, a shift that has raised frequent questions regarding their efficacy, tolerability, and cost-effectiveness [11]. The effects of protein supplementation on muscle mass and strength when combined with RT are already well established in the literature [22,23]. Morton and colleagues [2] previously concluded that protein supplementation combined with RT results in greater increases in fat-free mass and muscular strength compared to RT alone, with the effect being more pronounced in trained individuals and attenuated with advancing age.

Initially, it was hypothesized that whey protein would be superior in promoting gains in LBM due to its higher leucine content, which stimulates myofibrillar protein synthesis to a greater extent than plant-based proteins such as soy, or even casein, another animal-derived protein [24]. Lim et al. [25] conducted a meta-analysis reporting a favorable effect of animal protein on relative lean mass gain, although no significant differences were observed for absolute LBM, and RT did not appear to influence these results. However, that meta-analysis exhibited high heterogeneity because it did not account for differences in RT protocols and included diverse animal protein sources such as meat and milk. More reliable conclusions regarding supplementation effects should minimize dietary intake variability to isolate the true impact of high-protein supplementation, an approach adopted in the current study.

Messina et al. [26] reported no significant differences between WPS and soy protein supplementation for LBM improvement; both were effective in increasing muscle mass, with no statistically significant distinction between groups. Similarly, studies have indicated comparable effectiveness between PPS and WPS for maintaining lean mass in older adults [4,5]. Consistent findings were also reported for rice [27,28] and pea protein [29], as well as for plant-based protein blends rich in essential amino acids [30]. These results prompt reconsideration of the unique role of leucine in WPS, since plant-based proteins may elicit comparable effects on LBM [31].

RT appears to be more effective than protein supplementation alone in promoting adaptations in muscular strength [2], although supplementation can serve as complementary strategy to enhance RT outcomes. Nevertheless, the positive impact of WPS on LBM and BFM may depend on protein quality, particularly its amino acid composition [25], or fortification with compounds such as *β*-hydroxy *β*-methylbutyrate (HMB) and vitamin D [32]. Likewise, well-formulated PPS (such as combinations of pea, rice, hemp, and potato) have demonstrated the ability to stimulate myofibrillar protein synthesis comparably to WPS, including improved muscle function recovery up to 72 hours post-exercise [7]. Conversely, single-source plant-based protein formulations, particularly isolated hemp protein [33] or peanut protein [34], may yield less favorable results than multi-source blends. Thus, further research is needed to directly compare optimized multi-source plant-based protein formulations with single-source proteins to determine whether strategic amino acid blending is essential to maximize their biological value and hypertrophic potential.

Among the studies analyzed in this meta-analysis, Lynch et al. [21] suggested that approximately 2 g of leucine is required to stimulate muscle hypertrophy. Accordingly, they prescribed 19 g of whey protein isolate and 26 g of soy protein isolate to achieve this leucine threshold. Combined with RT, both interventions promoted LBM gains without significant differences between protein sources. It should be noted, however, that this leucine-matching approach was reported in only one of the five included studies, and therefore this finding should be interpreted with caution and cannot be generalized across all plant-based protein sources included in the present meta-analysis. In contrast, Mobley and coworkers [18] found that neither PPS nor WPS provided advantages over isolated leucine supplementation alone; however, WPS increased the number of satellite cells in skeletal muscle, a finding warranting further investigation.

Particular attention should be given to the study by Volek and co-authors [19], which compared soy and whey protein supplementation over a longer intervention period with higher total protein intake. After nine months of supplementation with 22 g/day combined with RT, WPS showed superior effects on LBM. The authors suggested that total daily protein intake may need to increase from 1.2 to 1.4 g/kg when using WPS to achieve optimal results. The duration of the intervention seems to be a key determinant of body composition changes, as evidenced by Zbinden-Foncea et al. [17], who evaluated RT effects combined with protein supplementation for only eight weeks. It is important to note that this represents the only study in the present meta-analysis using chia-based protein, and no significant changes in LBM were observed in that investigation. Therefore, the current evidence is insufficient to support conclusions regarding the efficacy of chia-based protein supplementation for body composition outcomes, and future dedicated trials are warranted. Additionally, although participants consumed 30 g of whey protein concentrate (providing 23 g of protein daily), no significant changes in BFM or maximal strength were observed, nor were there differences in the chia protein group. Therefore, intervention duration, regardless of the type of supplementation used, may represent a critical factor influencing body composition outcomes and should be carefully considered in future randomized trials.

Singh and contributors [20] conducted a complex study comparing varying dosages of whey and pea protein formulations under a high-frequency RT protocol (six weekly sessions over 12 weeks) alternating upper- and lower-body training. In this trial, WPS was not superior to PPS in promoting LBM gains, suggesting that, in addition to intervention duration, other factors may contribute to the discrepancies among studies, . These findings may also reflect other influential variables, including higher mean participant age, the use of lysine-rich pea protein, and unique RT strategies not previously examined in earlier meta-analyses.

Overall, the findings of this systematic review and meta-analysis, in contrast to previous investigations that also included trained individuals and may therefore have introduced methodological bias [10,32], suggest that PPS may support favorable adaptations in body composition during RT in untrained adults. Although WPS has traditionally been considered advantageous due to its amino acid profile and leucine content, the present findings indicate that PPS may also contribute to increases in LBM when total protein intake and training variables are adequately controlled.

However, the magnitude and consistency of these effects appear to depend on several factors, including protein source, amino acid composition, supplementation dosage, intervention duration, and participant characteristics. Therefore, while PPS represents a promising nutritional strategy for individuals who prioritize plant-based protein sources for ethical, environmental, economic, or gastrointestinal reasons, the current evidence remains insufficient to establish physiological equivalence with WPS across different training contexts and populations.

## 5. Conclusion

The findings of this systematic review and meta-analysis suggest that plant-based protein supplementation may support favorable changes in lean body mass and body composition in untrained adults engaged in resistance training. When combined with structured RT and adequate total protein intake, soy- and pea-based protein supplements may represent practical nutritional alternatives for individuals seeking non-animal-derived protein sources. Regarding chia-based protein, the available evidence is currently limited to a single study that did not demonstrate significant changes in lean body mass; therefore, firm conclusions regarding its efficacy cannot be drawn at this time.

Nevertheless, the limited number of available studies, methodological variability, and differences in supplementation protocols prevent definitive conclusions regarding equivalence between plant-based and whey protein supplementation. It should also be noted that the wide confidence intervals observed across studies, combined with the small number of included trials, substantially reduce the statistical power of the present meta-analysis, further precluding any conclusions regarding physiological equivalence. Furthermore, the search was restricted to three databases (PubMed, Web of Science, and ScienceDirect). This choice was pre-specified in our registered PROSPERO protocol, and PubMed and Web of Science together provide substantial overlapping coverage with databases such as CENTRAL and Embase for records indexed as randomized controlled trials, our mandatory study design criterion; nonetheless, the exclusion of CENTRAL, Scopus, Embase, and SPORTDiscus represents a limitation that may have affected the comprehensiveness of the evidence base, and future updates of this review should consider a broader search. Additionally, formal tests for publication bias (Begg’s and Egger’s tests) were not performed, as these methods have limited statistical power with fewer than ten studies and could produce misleading results in the context of the present meta-analysis. Sensitivity analysis through a leave-one-out approach was also deemed uninformative given the small number of included studies (four for BFM and five for LBM), as its application would have further reduced the precision of the pooled estimates. Therefore, future randomized controlled trials with longer intervention durations, larger sample sizes, standardized dosages, and more diverse participant profiles are warranted to strengthen evidence-based recommendations across different training contexts.

## Data Availability

All data included in this systematic review and meta-analysis were extracted from previously published studies. No new datasets were generated. Extracted data supporting the findings of this review are available from the corresponding author upon reasonable request.
